# Impact of Healthy Lifestyle Factors on Life Expectancy and Lifetime Health Care Expenditure: Nationwide Cohort Study

**DOI:** 10.2196/57045

**Published:** 2024-07-17

**Authors:** Wei-Cheng Lo, Tsuey-Hwa Hu, Cheng-Yu Shih, Hsien-Ho Lin, Jing-Shiang Hwang

**Affiliations:** 1 Master Program in Applied Epidemiology College of Public Health Taipei Medical University New Taipei Taiwan; 2 School of Public Health College of Public Health Taipei Medical University Taipei Taiwan; 3 Institute of Statistical Science Academia Sinica Taipei Taiwan; 4 Institute of Epidemiology and Preventive Medicine College of Public Health National Taiwan University Taipei Taiwan

**Keywords:** healthy lifestyle factors, life expectancy, lifetime health care expenditure, rolling extrapolation algorithm, nationwide cohort study, nonsmoking, moderate alcohol consumption, physical activity, fruit and vegetable intake, optimal body weight

## Abstract

**Background:**

The association between lifestyle risk factors and the risk of mortality and chronic diseases has been established, while limited research has explored the impact of healthy lifestyle factors on lifetime health care expenditure using longitudinal individual data.

**Objective:**

We aimed to determine the individual and combined effects of 5 healthy lifestyle factors on life expectancy and lifetime health care expenditure in Taiwan.

**Methods:**

Using data from the National Health Interview Survey cohort, 5 healthy lifestyle behaviors were defined and analyzed: nonsmoking, avoiding excessive alcohol consumption, engaging in sufficient physical activity, ensuring sufficient fruit and vegetable intake, and maintaining a normal weight. We used a rolling extrapolation algorithm that incorporated inverse probability of treatment weighting to estimate the life expectancy and lifetime health care expenditure of the study populations with and without healthy lifestyle factors.

**Results:**

A total of 19,893 participants aged ≥30 (mean age 48.8, SD 13.4) years were included, with 3815 deaths recorded during a median follow-up period of 15.6 years. The life expectancy and per capita estimated lifetime health care expenditures for the overall study population were 35.32 years and US $58,560, respectively. Multivariable-adjusted hazard ratios for all-cause mortality in participants adhering to all 5 healthy lifestyle factors, compared with those adhering to none, were 0.37 (95% CI 0.27-0.49). We found significant increases in life expectancy for nonsmokers (2.31 years; 95% CI 0.04-5.13; *P*=.03), those with sufficient physical activity (1.85 years; 95% CI 0.25-4.34; *P*=.02), and those with adequate fruit and vegetable intake (3.25 years; 95% CI 1.29-6.81; *P*=.01). In addition, nonsmokers experienced a significant reduction in annual health care expenditure (−9.78%; 95% CI −46.53% to −1.45%; *P*=.03), as did individuals maintaining optimal body weight (−18.36%; 95% CI −29.66% to −8.57%; *P*=.01). Overall, participants adhering to all 5 healthy lifestyle behaviors exhibited a life gain of 7.13 years (95% CI 1.33-11.11; *P*=.02) compared with those adhering to one or none, with a life expectancy of 29.19 years (95% CI 25.45-33.62). Furthermore, individuals adopting all 5 healthy lifestyle factors experienced an average annual health care expenditure reduction of 28.12% (95% CI 4.43%-57.61%; *P*=.02) compared with those adopting one or none.

**Conclusions:**

Adopting a healthy lifestyle is associated with a longer life expectancy and a reduction of health care expenditure in Taiwanese adults. This contributes to a more comprehensive understanding of the impact of healthy lifestyle factors on the overall health and economic burden.

## Introduction

### Background

Accumulative epidemiologic studies have investigated the relationship between lifestyle factors and the risk of mortality and chronic diseases. These studies have explored both the individual and combined effects of various risk factors on health. A meta-analysis including 531,804 participants from 17 countries indicated that smoking, inactivity, poor diet quality, and heavy alcohol consumption contributed to approximately 60% of all premature deaths [1]. A healthy lifestyle, which is characterized by regular physical activity, normal weight, nonsmoking behavior, moderate alcohol consumption, and healthy diet intake, is associated with an increase in life expectancy (LE) [2-11], with studies reporting increases of 6.6 years for men and 8.1 years for women in Singapore [2]; 8.8 years for men and 8.1 years for women in China [3]; 10.3 years for men and 8.3 years for women in Japan [4]; 12.2 years for men and 14.0 years for women in the United States [5]; 16.8 years for men and 18.9 years for women in Canada [6]; and 7.4 to 15.7 years for 3 European cohorts (RCPH, ESTHER, and Tromsø) [7], indicating that the beneficial effects of a healthy lifestyle may vary across populations and countries. On the other hand, these healthy lifestyle factors have been found to be associated with an increase in disease-free LE as well [12-17].

In recent decades, a significant increase has been noted in the average LE worldwide. However, whether such improvements translate into an overall reduction in health care expenditures remains uncertain. Studies on the impact of a healthy lifestyle on health care costs have focused primarily on the effects of individual risk behavior [18-28]. A regression analysis revealed that a 10% relative decrease in the prevalence of smoking in the United States would lead to a reduction of approximately US $63 billion in health care costs in the year following this decrease [21]. Another study reported that excessive alcohol consumption results in an economic burden of US $223.5 billion in the United States, with 72.2% of this amount being attributable to lost productivity, 11% attributable to health care expenses, 9.4% attributable to criminal justice costs, and 7.5% attributable to other effects [22]. A systematic review recruited 19 cost-of-illness studies on obesity, identifying substantial economic burdens across countries, with direct medical costs ranging from 0.7% to 17.8% of health system expenditure and total costs ranging from 0.05% to 2.42% of the country’s gross domestic product [23]. Studies comparing the effects of obesity, overweight, smoking, and problem drinking on health care use and costs in the United States have indicated that obesity and overweight are associated with the highest health care costs [26,27]. However, these studies had cross-sectional designs and certain analytical limitations, such as problems related to the unit of observation used at the ecological level or the indirect estimation of risk attributable approach. To date, no study has investigated the impact of both individual and combined healthy lifestyle factors on lifetime health care expenditure based on longitudinal individual data.

### Objectives

The objective of this study was to investigate the impact of both individual and combined healthy lifestyle factors on LE and lifetime health care expenditure in a contemporary population. Using a nationally representative cohort with over 19,000 participants, we have proposed a rolling extrapolation algorithm that incorporated inverse probability of treatment weighting (IPTW), which can adjust for the effects of potential confounders, to estimate the lifetime survival function for the study cohort. The lifetime survival functions were used to calculate the LE and lifetime health care expenditure for study populations with and without healthy lifestyle factors.

## Methods

### Study Design, Settings, and Population

**Figure 1:**
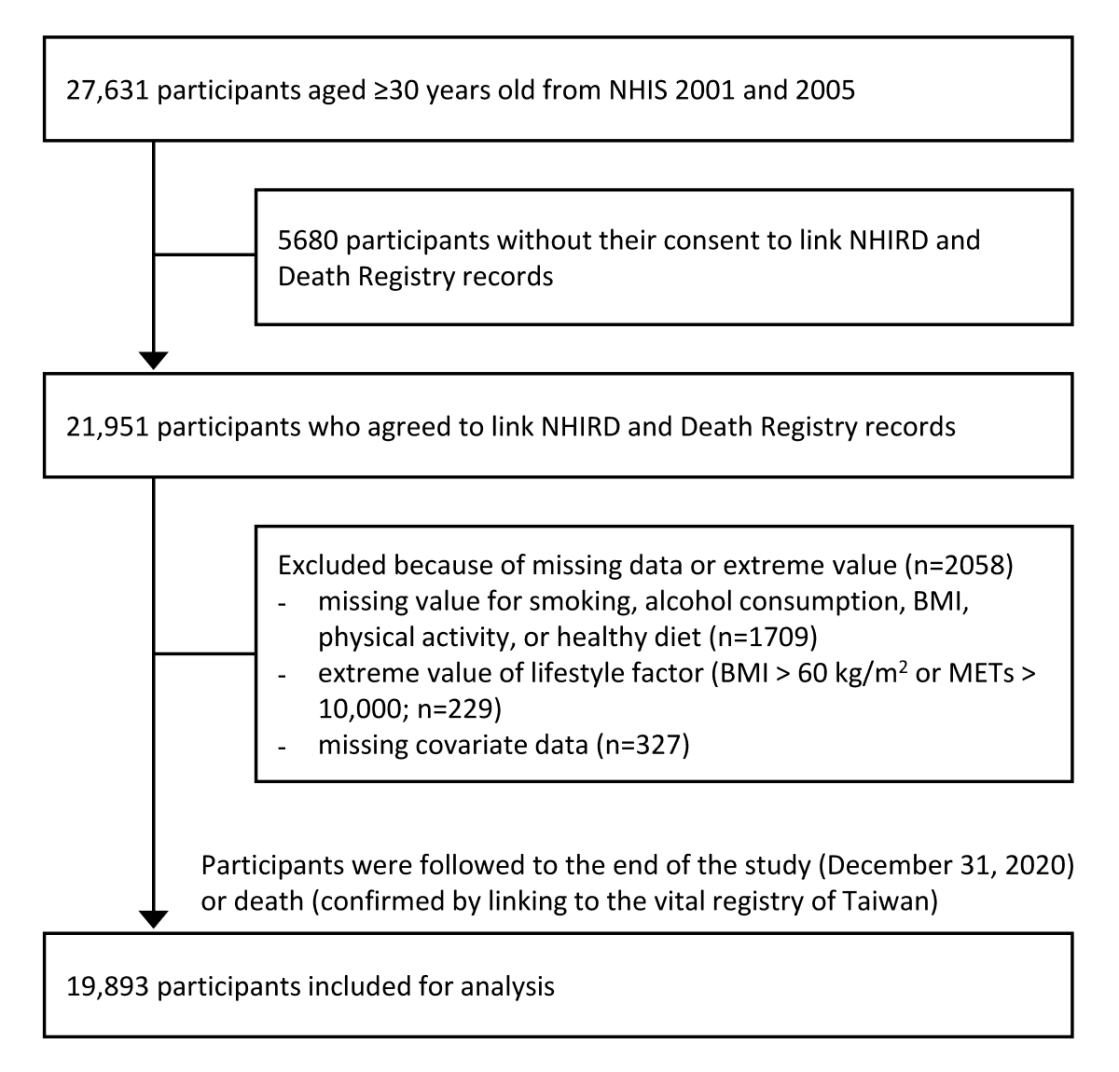
Flow diagram of participant selection. MET: metabolic equivalent of task; NHIRD: National Health Insurance Research Database; NHIS: National Health Interview Survey.

A nationwide longitudinal cohort study was performed. We used data from the Taiwan National Health Interview Survey (NHIS) to define study cohorts with or without healthy lifestyle factors. Then, a rolling extrapolation algorithm was introduced to estimate the lifetime survival function of study cohorts [29]. The LE of a study cohort was calculated according to the corresponding extrapolated lifetime survival function. In addition, the total lifetime health care expenditures of a study cohort were estimated by integrating the product of the lifetime survival function and a medical cost function, which was calculated using reimbursement data obtained from Taiwan’s National Health Insurance Research Database (NHIRD) [29]. Finally, we estimated the effect of each healthy lifestyle factor (individual effects) and the combination of the individual effects (combined effects) on LE and lifetime health care expenditure. The study sample comprised individuals who participated in the Taiwan NHIS in 2001 and 2005, with household response rates of 91.1% and 80.6%, respectively. The NHIS is a cross-sectional survey, which adopted a multistage stratified sampling scheme to obtain a nationally representative sample of the Taiwanese population. For the NHIS, baseline data regarding individuals’ sociodemographic and behavioral factors were collected through in-person interviews. Details of the design and sampling scheme of the NHIS have been reported previously [30]. In this study, 27,631 participants aged ≥30 years were included. We excluded participants who refused to link their NHIS data to NHIRD records (n=5680); who had a missing value for smoking, alcohol consumption, BMI, physical activity, or healthy diet (n=1709); who had an extreme value of lifestyle factor (BMI >60 kg/m^2^ or metabolic equivalent of task [MET]>10,000 min/wk; n=229); or who had missing covariate data (n=327). Finally, 19,893 participants were included in the analysis at baseline and were followed till the end of the study period (December 31, 2020) or death, which was confirmed using data from the vital registry of Taiwan (Figure 1). The reporting of this study adhered to the STROBE (Strengthening the Reporting of Observational Studies in Epidemiology) guidelines.

### Assessment of Healthy Lifestyle Factors

The following 5 lifestyle factors were analyzed in this study: smoking, alcohol consumption, BMI, leisure-time physical activity, and healthy diet (sufficient intake of fruit and vegetables). Information on lifestyle-related factors was collected from the NHIS data, which were collected through in-person interviews conducted at baseline. We categorized smoking status at baseline into 2 groups: never-smokers (never smoked at all or smoked <100 cigarettes in their lifetime) and current or former smokers (smoked more than 100 cigarettes in their lifetime). We defined alcohol consumption at baseline into 2 groups as well: infrequent or nonconsumers (those consuming alcohol less than once a week) and excess consumers (those consuming alcohol at least once a week). Anthropometric data were self-reported. We used the BMI to categorize baseline body weight status into 2 groups: optimal body weight (BMI: 18.5-25 kg/m^2^) and nonoptimal body weight (underweight: BMI <18.5 kg/m^2^ and overweight: BMI ≥25 kg/m^2^). To evaluate leisure-time physical activity, we used individuals’ responses to the following NHIS question: Did you participate in any leisure-time physical activity over the past 2 weeks? Respondents could list more than one type of physical activity; they also reported the exercise frequency (ie, the number of times they engaged in the activity in the previous 2 weeks) and duration (ie, how long they exercised). MET intensity levels were assigned to each activity (eg, Tai Chi, walking, jogging, and swimming) on the basis of a relevant study [31]. Total weekly leisure-time physical activity (MET min/wk) was calculated by multiplying the frequency (times per week), duration (minutes), and the MET values of each activity. The physical activity was classified into 2 groups: insufficiently active (<500 MET min/wk) and sufficiently active (≥500 MET min/wk). Diet was assessed using the NHIS data, which were collected using a simplified food frequency questionnaire. We defined a healthy diet as having sufficient fruit and vegetable intake: ≥4 servings of fruits and vegetables per week.

### Covariates

We obtained data regarding the individuals’ sociodemographic characteristics, lifestyle factors, and disease history from the NHIS database. The NHIS data were obtained through in-person interviews and a structured questionnaire survey [30]. The potential confounders and covariates were taken into consideration, including enrollment year, age at baseline, sex, ethnicity, education level, marital status, religion, monthly household income, and medical history of hypertension, hyperlipidemia or raised cholesterol, cardiovascular disease, diabetes mellitus, cancer, chronic lung diseases (asthma or chronic obstructive pulmonary disease), and chronic kidney diseases (Table S1 in Multimedia Appendix 1).

### Statistical Analysis

Although the participants of the study cohort were followed with a median duration of 15.6 years, a high proportion of participants were still alive by the end of the follow-up. We therefore applied a rolling extrapolation algorithm to obtain the lifetime survival function for the study cohort [29]. The rolling extrapolation algorithm is one of the new methods to improve the accuracy of survival extrapolation using external evidence [32]. The extrapolation method was successfully applied in many studies for estimating loss of LE of cohorts with specific health conditions [33-36] compared with age- and sex-matched reference cohorts generated from life tables of the general population. In an observational study, the estimates of LE differences among comparative study cohorts (eg, with and without specific healthy lifestyle factors) may be biased due to the effects of some confounders in the samples. In this study, we incorporated IPTW to the rolling extrapolation algorithm to reduce the potential confounding effects on the estimate of LE difference between 2 cohorts.

We described the details of the modified rolling extrapolation algorithm in the following 4 major steps. First, each individual in a study cohort was given a weight equal to the inverse probability of the individual being in the cohort, which is estimated by a multinomial logistic regression model with measured confounding covariates; that is, each study cohort is inflated with the weights of the participants to form a pseudocohort in which confounders are equally distributed across the pseudocohorts [37]. The adjusted Kaplan-Meier estimator was applied to the weighted survival data of individuals in the study cohort to obtain the survival function, denoted by *S*(*t*), for the cohort [38]. We may call *S*(*t*) the confounder-adjusted survival function of the study cohort. Second, Monte Carlo methods were used to generate survival times of referents whose age and sex matched with participants in the study cohort using life tables of the general population. The same weights were assigned for the referents matched with the participants in the study cohort. The confounder-adjusted lifetime survival function of the reference population was obtained by applying an adjusted Kaplan-Meier estimator on the weighted survival times of the generated referents and denoted by *S_r_*(*t*). We renamed *S_r_(t)* as *S_p_(t)* for use as a relevant predictor in extrapolating the survival function of the study cohort when *S_r_(t)>S(t)* during the follow-up period. If *S_r_(t) ≤ S(t)*, a proper hazard value δ was subtracted from the reference population to ensure the predictor *S_p_(t)=S_r_(t)×e^δt^>S(t)* [39]. The relative survival function, 
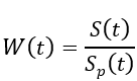
, is then within 0 and 1. Third, the logit transformation of the relative survival would straighten the curve logit[W(t)], which is relatively easy to extrapolate. Fourth, we used restricted cubic spline models to fit logit[*W*(t)] for the observed period *t=1,2,...,F* months. The fitted curve was used to predict the logit[*W*(t)] 1 month ahead. The predicted logit[*W*(F+1)] was usually quite accurate because of the approximate linearity property of logit[*W*(t)] and treated as an *observation* at month F+1. We then repeated the extrapolation procedures by rolling the same-length observation periods 1 month ahead, *t=2,3,...,F+1*, and refit the restricted cubic spline models for the updated observation periods to predict the value of logit[*W*(F+2)]. By repeatedly performing the abovementioned procedures of extrapolating logit[*W*(t)] month-by-month to a time *L* beyond which all subjects of the cohort died, we could then invert transformation of the extrapolated logit[W(t)] to obtain an estimate of relative survival function 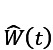 and lifetime survival function 
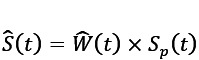
 of the cohort. The validation analysis of the rolling extrapolation algorithm was performed for study cohorts with and without risk lifestyle factors (Table S2 in Multimedia Appendix 1). The confounder-adjusted LE of each study cohort was obtained by summing the extrapolated confounder-adjusted lifetime survival function, 
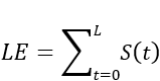
. The loss or gain of LE of a cohort is estimated by the difference of confounder-adjusted LE between the cohort and the comparative cohort.

With the extrapolated lifetime survival function S(t) of a cohort, we can further estimate the lifetime health expenditures of the cohort using the formula 
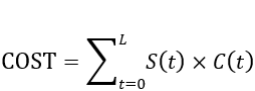
 where C(t) is monthly average expenditures of living participants at time *t* in the study cohort [29]. We retrieved all direct health care costs (including inpatient and outpatient care) from the claims database of the National Health Insurance for the participants of each study cohort. Each participant’s costs were also assigned the same weight as the participant in a cohort. All the reimbursement costs, including treatment, examination, and procedures associated with disease outcomes of the participants in a cohort, were weighted and summed up to calculate the monthly average of the cohort. To extrapolate the monthly mean cost function beyond follow-up, we make one reasonable assumption that medical expenditures start increasing from K months before death [40]. Specifically, we classify the participants alive in each month into subsets of participants who died in the current month, within the next K months, and who lived more than K months. We then calculate mean costs in each month by a weighted average of the K+2 mean costs of these subsets in that month, where the weights were estimated by the extrapolated risk of death and the mean cost of each subset was estimated by the average of the participants’ weighted expenditures in the subset [29]. Estimates of LE, difference in LE between study cohorts, lifetime health expenditures, annual average health expenditure, and their SEs and 95% CIs were obtained using the R package iSQoL2, which can be downloaded [41].

To estimate the magnitude of hazards associated with an unhealthy lifestyle, we used Cox proportional hazard models to calculate adjusted hazard ratios and corresponding 95% CIs for all-cause mortality associated with each individual health risk factor and a composite of health risk factors (0, 1, 2, 3, 4, or 5). The models were adjusted for the effects of the aforementioned covariates. In addition, dose-response relationships between the lifestyle factors and LE or health care expenditure were analyzed to evaluate the robustness of our findings. We classified smoking status into 4 categories based on smoking history and recent smoking behavior: never-smokers, former smokers, current smokers of 1 to 19 cigarettes per day, and current smokers of ≥20 cigarettes per day. Alcohol consumption was classified into 4 categories based on drinking frequency: never drinkers, infrequent drinkers (those consuming alcohol less than once a week), regular drinkers (those consuming alcohol more than once a week but not to the point of getting drunk), and heavy drinkers (those consuming alcohol more than once a week and typically to the point of getting drunk). Leisure-time physical activity was divided into 4 categories: sedentary (0 MET min/wk), insufficiently active (1-499 MET min/wk), active (500-1499 MET min/wk), and highly active (≥1500 MET min/wk). We classified healthy diet into 4 categories based on the frequency of fruit and vegetable intake: ≤1 serving, 2 to 3 servings, 4 to 6 servings, and ≥7 servings per week. Body weight status was divided into 4 categories: underweight (BMI <18.5 kg/m^2^), normal weight (18.5≤BMI<25 kg/m^2^), overweight (25≤BMI<30 kg/m^2^), and obese (BMI ≥30 kg/m^2^) [42]. Moreover, a series of sensitivity and validation analyses were conducted. We used the World Health Organization (WHO) Asian BMI risk cut points for BMI classification as part of our sensitivity analysis [43]. To investigate the effect of the number of healthy lifestyle factors on health, we initially designated individuals who adhered to none or one of the 5 healthy lifestyle factors as the reference group in our primary analysis. As a supplementary sensitivity analysis, we also designated individuals who embraced none of the healthy lifestyle factors as an alternative reference group. Furthermore, a subgroup analysis was performed based on different numbers of healthy lifestyle factors, including those with 2 factors, 3 factors, or 4 factors, to present estimations of LE and health care expenditure associated with the combinations of healthy lifestyle factors within each subgroup. In addition, a validation analysis was performed to assess the accuracy of survival function extrapolation (further details are provided in Table S2 in Multimedia Appendix 1). The R package programs nnet::multinom and survival::coxph were used for the analysis. Statistical significance was set at a 2-tailed value of *P*<.05.

### Ethical Considerations

In accordance with Article 5 of the Human Subjects Research Act of the Ministry of Health and Welfare, Republic of China (Taiwan) [44] and the guidelines titled “Management Principles for the Application of Health and Welfare Data” announced by the Ministry of Health and Welfare, Taiwan on September 7, 2017, pursuant to Wei-Bu-Tong-Zi (#1062560770)” [45], the data used in this analysis are subject to the management and regulation by the Health and Welfare Data Science Center, Taiwan [46]. Individual health data managed therein (including NHIS, NHIRD, and Death Registry data) undergo deidentification processing in accordance with the Personal Data Protection Act.

This study was approved by the Taipei Medical University Joint Institutional Review Board (TMU-JIRB-N202009047). The Health and Welfare Data Science Center reviewed the study protocol and institutional review board approval and granted access to confidential data. The requirement for written informed consent was therefore waived because anonymous and deidentified information was used for analysis.

## Results

### Baseline Characteristics

Of 19,893 participants included in the analysis, 10,311 (51.8%) were men; the mean age was 48.8 (SD 13.4) years. During 317,116 person-years of follow-up, 3815 deaths were recorded (1403 women and 2412 men). Table 1 presents the baseline characteristics of study participants by the number of healthy lifestyle behaviors they adhered to. Individuals adhering to a higher number of healthy lifestyle behaviors were more likely to be women, had received higher levels of education, and had higher household income. At baseline, 68.2% of participants were not current or former smokers, 83.2% did not consume excessive amounts of alcohol, 33.8% were sufficiently active, 89.2% had sufficient fruit and vegetable intake, and 62.1% had a normal weight.

**Table 1:** Baseline demographic and lifestyle characteristics of the study population according to a number of healthy lifestyle factors.

				Healthy lifestyle factors								
				0 or 1		2		3		4		5
**Characteristics**												
	Age (y), mean (SD; range)			45.7 (11.6; 30-90)		47.6 (13.0; 30-96)		49.1 (13.6; 30-98)		48.8 (13.7; 30-98)		50.9 (13.3; 30-93)
	**Sex, n (%)**											
		Male	1121 (91.89)		2484 (84.81)		3297 (57.36)		2526 (35.17)		883 (31.39)	
		Female	99 (8.11)		445 (15.19)		2451 (42.64)		4657 (64.83)		1930 (68.61)	
	**Education, n (%)**											
		Less than elementary school	384 (31.48)		947 (32.33)		2114 (36.78)		2351 (32.73)		796 (28.30)	
		High school	718 (58.85)		1551 (52.95)		2542 (44.22)		3158 (43.96)		1183 (42.05)	
		College or above	118 (9.67)		431 (14.71)		1092 (19.00)		1674 (23.31)		834 (29.65)	
	**Marriage, n (%)**											
		Married or cohabiting	856 (70.16)		2157 (73.64)		4335 (75.42)		5594 (77.88)		2192 (77.92)	
		Never married	171 (14.02)		323 (11.03)		528 (9.19)		506 (7.04)		199 (7.07)	
		Others^a^	193 (15.82)		449 (15.33)		885 (15.40)		1083 (15.08)		422 (15.00)	
	**Ethnic group, n (%)**											
		Minnan	798 (65.41)		1973 (67.36)		4134 (71.92)		5207 (72.49)		2028 (72.09)	
		Hakka	135 (11.07)		378 (12.91)		741 (12.89)		940 (13.09)		387 (13.76)	
		Mainland Chinese	94 (7.70)		302 (10.31)		537 (9.34)		800 (11.14)		341 (12.12)	
		Aborigines	182 (14.92)		252 (8.60)		270 (4.70)		151 (2.10)		29 (1.03)	
		Others	11 (0.90)		24 (0.82)		66 (1.15)		85 (1.18)		28 (1.00)	
	**Household income^b^, n (%)**											
		Low	307 (25.16)		672 (22.94)		1228 (21.36)		1422 (19.80)		573 (20.37)	
		Median	688 (56.39)		1575 (53.77)		3059 (53.22)		3702 (51.54)		1304 (46.36)	
		High	225 (18.44)		682 (23.28)		1461 (25.42)		2059 (28.66)		936 (33.27)	
	**Comorbid diseases, n (%)**											
		Hypertension	240 (19.67)		574 (19.60)		1252 (21.78)		1308 (18.21)		527 (18.73)	
		Hyperlipidemia or raised cholesterol	237 (19.43)		530 (18.09)		1083 (18.84)		1162 (16.18)		478 (16.99)	
		Cardiovascular disease	109 (8.93)		282 (9.63)		778 (13.54)		927 (12.91)		393 (13.97)	
		Stroke	27 (2.21)		87 (2.97)		214 (3.72)		203 (2.83)		84 (2.99)	
		Diabetes mellitus	84 (6.89)		237 (8.09)		481 (8.37)		545 (7.59)		210 (7.47)	
		Chronic kidney disease	57 (4.67)		162 (5.53)		294 (5.11)		313 (4.36)		103 (3.66)	
		Cancer	29 (2.38)		102 (3.48)		290 (5.05)		471 (6.56)		228 (8.11)	
		Asthma or chronic obstructive pulmonary disease	108 (8.85)		261 (8.91)		484 (8.42)		539 (7.50)		169 (6.01)	
**Lifestyle factors, n (%)**												
	**Cigarette smoking**											
		Never-smokers	59 (4.84)		617 (21.07)		3571 (62.13)		6515 (90.70)		2813 (100)	
		Current or former smokers	1161 (95.16)		2312 (78.93)		2177 (37.87)		668 (9.30)		0 (0)	
	**Alcohol consumption**											
		Infrequent or nonconsumers	213 (17.46)		1541 (52.61)		5002 (87.02)		6978 (97.15)		2813 (100)	
		Excess consumers	1007 (82.54)		1388 (47.39)		746 (12.98)		205 (2.85)		0 (0)	
	**Leisure-time physical activity**											
		Sufficient active	36 (2.95)		337 (11.51)		1025 (17.83)		2508 (34.92)		2813 (100)	
		Insufficient active	1184 (97.05)		2592 (88.49)		4723 (82.17)		4675 (65.08)		0 (0)	
	**Fruit and vegetable intake**											
		Sufficient intake	505 (41.39)		2194 (74.91)		5152 (89.63)		7075 (98.50)		2813 (100)	
		Low or insufficient intake	715 (58.61)		735 (25.09)		596 (10.37)		108 (1.50)		0 (0)	
	**BMI group**											
		Normal weight (BMI: 18.5-25 kg/m^2^)	221 (18.11)		1169 (39.91)		2494 (43.39)		5656 (78.74)		2813 (100)	
		Underweight (BMI <18.5 kg/m^2^)	84 (6.89)		141 (4.81)		439 (7.64)		154 (2.14)		0 (0)	
		Overweight (BMI ≥25 kg/m^2^)	915 (75.00)		1619 (55.27)		2815 (48.97)		1373 (19.11)		0 (0)	

^a^Others: widowed, divorced, separated, or serving as a single parent.

^b^Household income (US $ per month): low: <980; median: 980-3260; high: >3260.

### Healthy Lifestyle Factor and Mortality Risk

Each individual healthy lifestyle factor was significantly associated with all-cause mortality (Figure S1 and Table S3 in Multimedia Appendix 1). The risk of mortality decreased with increasing adherence to healthy lifestyle behaviors (in terms of the number of behaviors adopted by the individuals). Individuals adhering to all 5 healthy lifestyle behaviors exhibited a hazard ratio of 0.37 (95% CI 0.27-0.49) for all-cause mortality, compared with those with 0 healthy factors, after adjusting for potential confounding factors.

### Impact on LE

Using the rolling extrapolation method, we found that participants with mean ages of 48.8 years who did not smoke had a life gain of 2.31 (95% CI 0.04-5.13) additional years. Not drinking excessively was marginally associated with an additional gain in life years by 1.62 (95% CI −0.15 to 4.59) years. Sufficient physical activity was associated with an additional 1.85 (95% CI 0.25-4.34) life years gain. Sufficient fruit and vegetable intake was associated with a life gain of 3.25 (95% CI 1.29-6.81) years. Finally, having an optimal weight was associated with a nonsignificant increment in life years (0.72 years; 95% CI −1.21 to 2.14) compared with being underweight or overweight. LE increased with the increasing adherence to healthy lifestyle behaviors (Table 2). After covariate adjustment, we note that individuals who adhere to 4 and 5 healthy lifestyle behaviors have a life span increase of 6.58 (95% CI 1.13-10.76) and 7.13 (95% CI 1.33-11.11) years, respectively, compared with LE (29.19 years) for individuals who do not adhere to more than one healthy lifestyle behavior.

**Table 2:** Life expectancy and years of life gained of study cohorts with and without healthy lifestyle factors.

Lifestyle factors		Values, n	Life expectancy (95% CI)	Years of life gained (95% CI)
**Cigarette smoking**				
	Current or former smokers	6318	32.13 (29.47 to 34.12)	Reference
	Never-smokers	13,575	34.44 (33.17 to 35.81)	2.31 (0.04 to 5.13)
**Alcohol consumption**				
	Excess consumers	3346	33.43 (30.88 to 35.27)	Reference
	Infrequent or nonconsumers	16,547	35.05 (34.17 to 35.86)	1.62 (−0.15 to 4.59)
**Leisure-time physical activity**				
	Insufficient active	13,174	33.50 (31.89 to 34.29)	Reference
	Sufficient active	6719	35.35 (33.12 to 36.97)	1.85 (0.25 to 4.34)
**Fruit and vegetable intake**				
	Low or insufficient intake	2154	31.47 (28.72 to 33.56)	Reference
	Sufficient intake	17,739	34.72 (34.33 to 36.16)	3.25 (1.29 to 6.81)
**BMI group**				
	Nonoptimal body weight^a^	7540	32.86 (31.58 to 33.85)	Reference
	Optimal body weight	12,353	33.58 (32.14 to 34.22)	0.72 (−1.21 to 2.14)
**Number of low-risk lifestyle factors**				
	0 or 1	1220	29.19 (25.45 to 33.62)	Reference
	2	2929	30.69 (26.25 to 32.76)	1.50 (−5.28 to 6.36)
	3	5748	34.42 (33.82 to 36.04)	5.24 (0.98 to 9.70)
	4	7183	35.77 (34.27 to 36.83)	6.58 (1.13 to 10.76)
	5	2813	36.32 (32.76 to 38.42)	7.13 (1.33 to 11.11)

^a^Nonoptimal weight: underweight (BMI <18.5 kg/m^2^) and overweight (BMI ≥25 kg/m^2^).

### Impact on Lifetime Health Care Expenditure

We evaluated the effects of healthy lifestyle factors on lifetime health care expenditure and found that nonsmoking and a healthy weight were significantly associated with reduced lifetime health care expenditure (Table 3). Per capita estimated lifetime health care expenditures were US $60,395 for smokers and US $58,821 for nonsmokers. Notably, individuals with nonoptimal body weight had a significantly higher per capita estimated lifetime health care expenditure compared with those with optimal body weight (US $62,474 vs US $53,336, respectively). The percentage change in annual health care expenditure was calculated by determining the difference in per capita annual health care expenditure between populations with and without healthy lifestyle factors, which was then divided by the annual average health care expenditure per capita of the overall population. We found that never-smokers had a significant reduction in the percentage change in annual health care expenditure compared with current or former smokers (−9.78%; 95% CI −46.53% to −1.45%). Furthermore, individuals with optimal body weight had notably lower average annual health care expenditures compared with those with nonoptimal body weight, with an 18.36% reduction of annual health care expenditures per capita (95% CI 8.57%-29.66%). Overall, individuals who adopted 2, 3, 4, or 5 healthy lifestyle factors demonstrated a dose-response reduction in the percentage change in annual health care expenditure per capita by 16.01%, 18.90%, 18.61%, and 28.12%, respectively, in comparison to those who adopted 0 or only 1 healthy lifestyle factor.

**Table 3:** Lifetime health care expenditure and percentage change in annual health care expenditure of study cohorts with and without healthy lifestyle factors.

Lifestyle factors		Values, n	Lifetime health care expenditure, US $^a^ (95% CI)	Percentage change in annual health care expenditure, (%; 95% CI)^b^
**Cigarette smoking**				
	Current or former smokers	6318	60,395 (56,018 to 68,054)	Reference
	Never-smokers	13,575	58,821 (38,595 to 63,525)	−9.78 (−46.53 to −1.45)
**Alcohol consumption**				
	Excess consumers	3346	57,849 (51,399 to 65,353)	Reference
	Infrequent or nonconsumers	16,547	57,737 (54,723 to 60,531)	−5.00 (−19.14 to 6.11)
**Leisure-time physical activity**				
	Insufficient active	13,174	56,733 (53,081 to 59,840)	Reference
	Sufficient active	6719	60,828 (56,098 to 67,518)	1.59 (−7.46 to 11.14)
**Fruit and vegetable intake**				
	Low or insufficient intake	2154	52,815 (46,193 to 59,625)	Reference
	Sufficient intake	17,739	60,217 (58,016 to 63,905)	3.24 (−11.98 to 17.51)
**BMI group**				
	Nonoptimal body weight^c^	7540	62,474 (56,549 to 68,844)	Reference
	Optimal body weight	12,353	53,336 (49,394 to 55,884)	−18.36 (−29.66 to −8.57)
**Number of low-risk lifestyle factors**				
	0 or 1	1220	58,715 (43,229 to 78,470)	Reference
	2	2929	53,414 (45,675 to 58,903)	−16.01 (−46.06 to 12.47)
	3	5748	58,246 (40,663 to 63,068)	−18.90 (−46.53 to −1.70)
	4	7183	60,667 (40,685 to 67,255)	−18.61 (−47.41 to −1.84)
	5	2813	55,785 (47,896 to 64,346)	−28.12 (−57.61 to −4.43)

^a^US $1=New Taiwan $30.65.

^b^The percentage change in annual health care expenditure was calculated as the difference in per capita annual health care expenditure between populations with and without healthy lifestyle factors divided by the annual average health care expenditure per capita for the overall population.

^c^Nonoptimal weight: underweight (BMI <18.5 kg/m^2^) and overweight (BMI ≥25 kg/m^2^).

### Dose-Response Analysis

**Figure 2:**
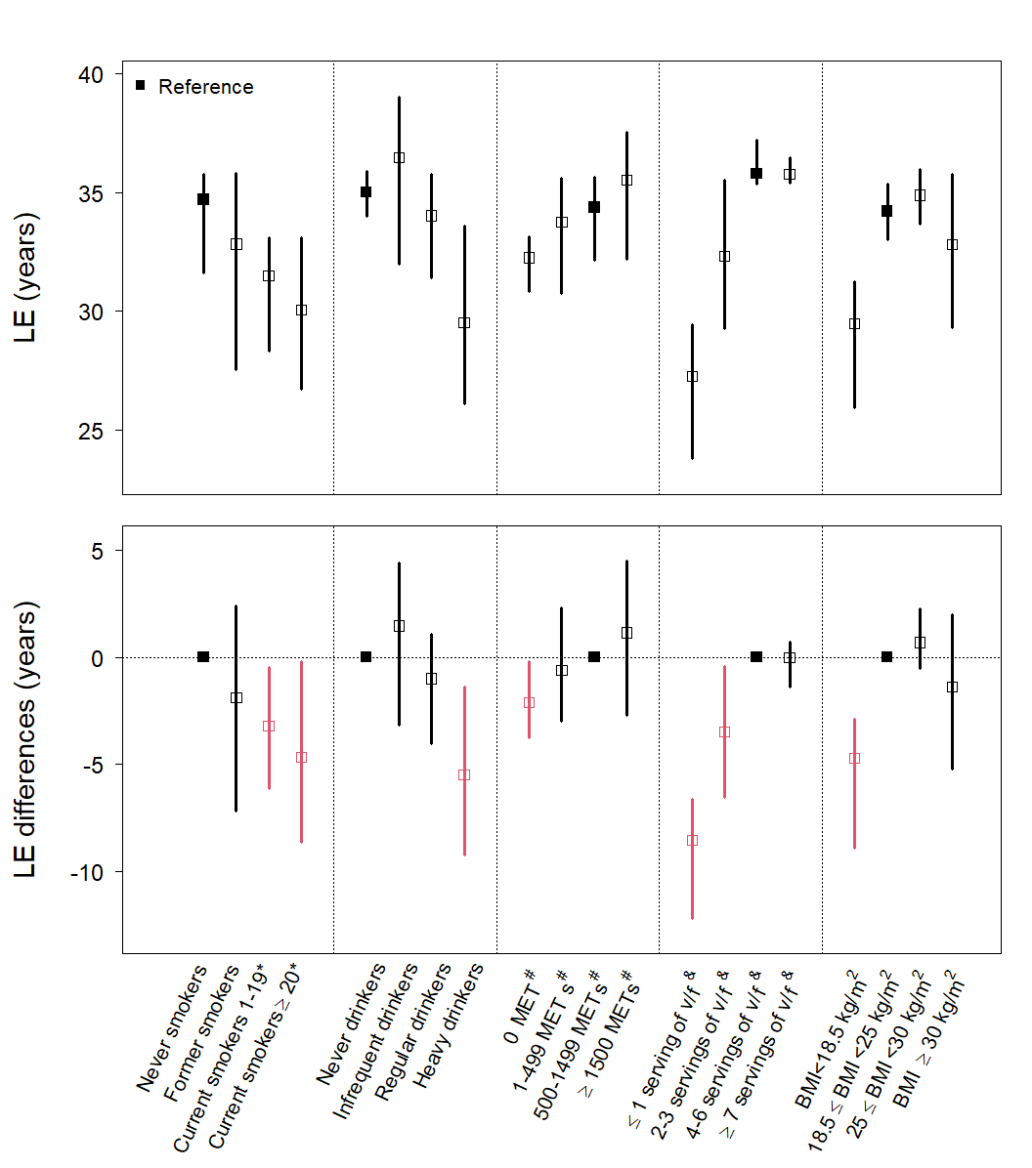
Estimated life expectancy (LE) and years of life gained according to levels of individual lifestyle risk factors. Red color indicates the estimated value was significantly higher or lower than reference group. MET: metabolic equivalent of task. *: cigarettes per day; #: minutes per week; &: servings of fruit and vegetable intake per week.

**Figure 3:**
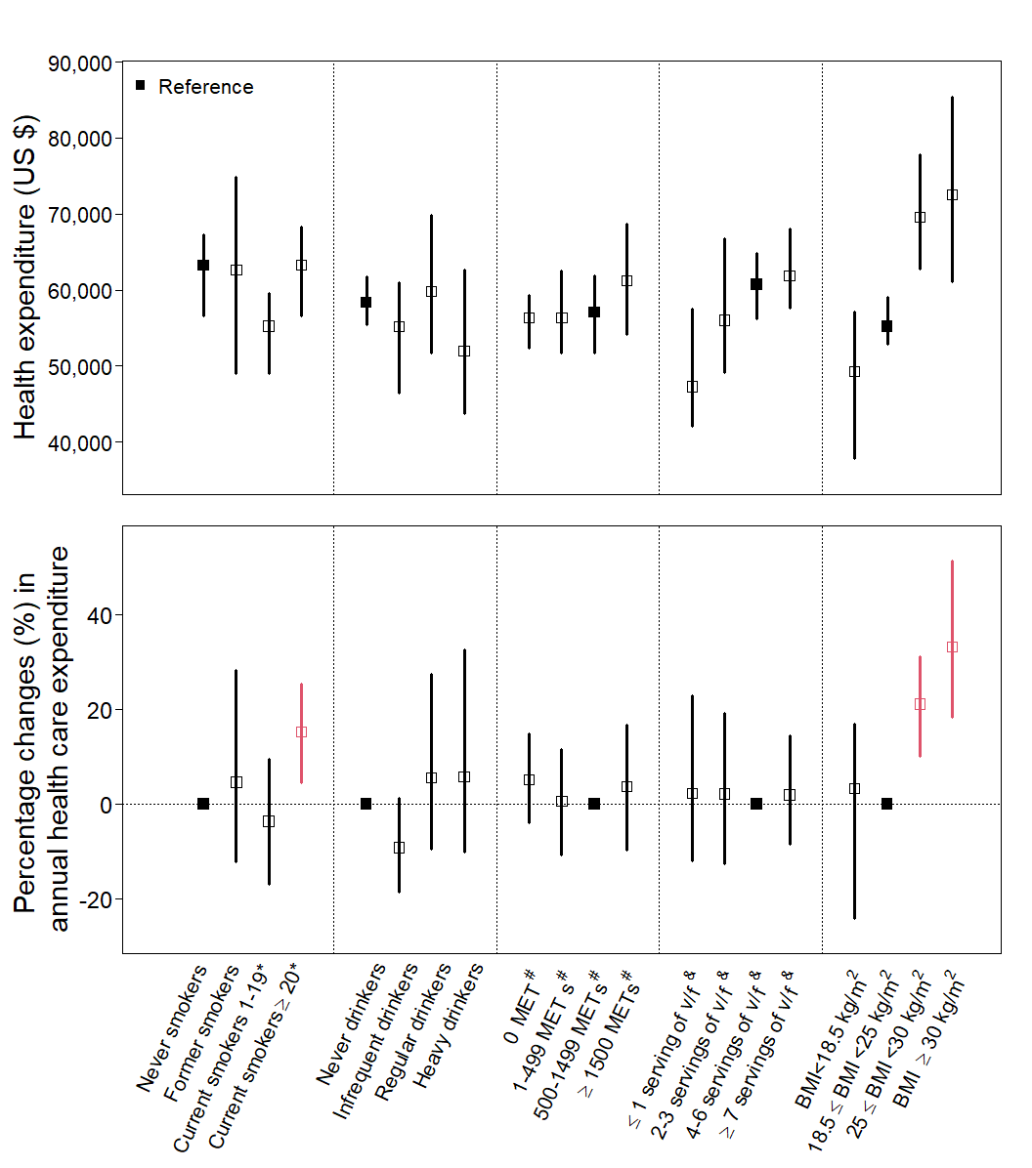
Estimated lifetime health care expenditure and percentage changes in annual average health care expenditure according to levels of individual lifestyle risk factors. MET: metabolic equivalent of task. Red color indicates the estimated value was significantly higher or lower than reference group. *: cigarettes per day; #: minutes per week; &: servings of fruit and vegetable intake per week.

The analysis of the dose-response effect of each lifestyle factor on LE revealed that nonsmoking, infrequent alcohol consumption (less than once a week), high physical activity levels, healthy diet intake (intake of ≥4 servings of fruits and vegetables per week), and overweight (25≤BMI<30 kg/m^2^) were associated with a long LE (Figure 2). Regarding lifetime health care expenditure, individuals who were heavy smokers (≥20 cigarettes per day), were regular and heavy drinkers, had a sedentary lifestyle (0 MET min/wk), and were overweight and obese (BMI ≥25 kg/m^2^) are more likely to have higher average annual health care expenditures (Figure 3). Regarding healthy diet intake, it is important to note that the exposure group with the longest LE may not necessarily be the same as the group with the lowest health care expenses. In addition, we found that overweight, when compared with normal weight, was associated with an additional 0.69 (95% CI −0.54 to 2.26) life years. However, this increased LE comes with the caveat of incurring additional health care expenses, with an increase of percentage change in annual health care expenditure (21.1%; 95% CI 9.98%-31.17%). Alternatively, we depicted the correlation between LE and lifetime health care expenditures for populations with and without healthy lifestyle factors using a scatter plot. We hypothesized a linear relationship, with a steeper slope indicating higher annual average health care expenditures. Figure S2 in Multimedia Appendix 1 illustrates that, in comparison to populations with 0 or only 1 healthy lifestyle factor, individuals with all 5 healthy lifestyle factors not only experience a longer LE but also have lower annual average health care costs. When examining individual factors, generally, each of the healthy lifestyle factors is associated with a longer LE. However, we found that smoking and nonoptimal body weight are linked to considerably higher annual health care expenses, along with reduced LE.

### Sensitivity and Subgroup Analysis

In addition to using the globally common BMI classification criteria, we conducted a sensitivity analysis by incorporating the WHO Asian BMI risk cut points for BMI classification. The results of the sensitivity analysis align with our primary findings. Compared with individuals with a normal weight (18.5≤BMI<23 kg/m^2^), those classified as overweight (23≤BMI<27.5 kg/m^2^) demonstrated significantly improved LE, with an additional gain of 3.36 years (95% CI 1.88-5.76; Figure S3 in Multimedia Appendix 1). Moreover, we also designated individuals who adhered to none of the healthy lifestyle factors as an alternative reference group. A discernible dose-response relationship was identified. Overall, individuals who adopted 1, 2, 3, 4, or all 5 healthy lifestyle factors presented LE gains of 4.37, 4.73, 10.22, 11.85, and 12.39 years, respectively, in comparison to those who adopted none of these 5 healthy lifestyle factors (Table S4 in Multimedia Appendix 1). Furthermore, under stratification by the number of healthy lifestyle factors, noticeable heterogeneity emerged across various combinations of these factors. Among dual combinations of healthy lifestyle factors, the combination of nonsmoking and sufficient fruit and vegetable intake exhibited a higher life years gain (5.49, 95% CI −4.21 to 12.17). Within combinations of 3 healthy lifestyle factors, the combination of nonsmoking, sufficient fruit and vegetable intake, alongside maintaining an optimal body weight, yielded the most favorable outcome (life years gain: 10.64, 95% CI 5.77-14.83). Meanwhile, among combinations of 4 healthy lifestyle factors, the amalgamation of nonsmoking, moderate alcohol consumption, sufficient fruit and vegetable intake, and maintaining an optimal body weight was deemed optimal (life years gain: 9.91, 95% CI 5.51-14.07). These results emphasize the potency of the combination of nonsmoking and adequate consumption of fruits and vegetables in enhancing LE. In addition, regarding lifetime health expenditure, upholding an optimal body weight remains the paramount determinant, as combinations featuring the optimal weight factor distinctly curtail medical expenses (Table S5 in Multimedia Appendix 1).

## Discussion

### Principal Findings

Using data from a nationally representative cohort, we found that 5 healthy lifestyle factors were associated with significant gains in LE and reductions in health care expenditure for individuals aged ≥30 years in Taiwan. Our findings revealed that adherence to all 5 healthy lifestyle behaviors was associated with a 7.13-year increase in LE and a 28.12% reduction in the percentage of annual health care expenditure per capita compared with the values noted in individuals adhering to 0 or 1 healthy lifestyle behavior. Furthermore, the lifestyle factors did not exert equal effects on LE. Smoking and diet were the most significant risk factors for a short LE, with current and former smokers having an estimated reduction of 2.31 years in LE compared to never-smokers. In addition, individuals consuming sufficient amounts of fruits and vegetables had a life gain of 3.25 years compared with the LE of individuals with insufficient intake. Individuals regularly engaging in sufficient physical activity had a life gain of 1.85 years compared with the LE of individuals with a sedentary or inactive lifestyle. Individuals who were underweight (BMI <18.5 kg/m^2^) had a shorter LE than did those with a normal weight (18.5≤BMI<25 kg/m^2^); in contrast, individuals who were overweight (25≤BMI<30 kg/m^2^) had a longer LE than did those with a normal weight. Heavy drinkers who consumed alcohol more than once a week and typically to the point of getting drunk had a shorter LE than those consuming no alcohol.

Our findings regarding the individual and combined effects of healthy lifestyle factors on LE are generally consistent with those of other studies. Adhering to a healthy lifestyle is associated with a significant increase in estimated LE by 6.6 to 18.9 years across countries [2-8]. Differences in the definitions of healthy lifestyles and the characteristics of study populations might have contributed to the differences in LE increase among the countries. Additional sources of such heterogeneity should be investigated. For example, living environment has also been linked to LEs [47-49]. Residents of low-income areas in wealthy cities, such as New York and San Francisco, had significantly longer LEs than did those of low-income areas in poor cities, such as Gary, Indiana, and Detroit [49]. Further studies are required to better understand how living environment, as well as socioeconomic status and difference between Eastern and Western populations, influence the health benefits of lifestyle factors across countries.

The global average LE has undergone a significant increase in recent decades. However, the LE in Taiwan, one of the wealthiest nations in East and Southeast Asia, falls behind in comparison to other high-income countries in this region. In 2019, LE at birth in Taiwan was 80.3 years, which was lower than that of Singapore (84.9 years), Japan (84.8 years), and South Korea (82.9 years) [50,51]. This discrepancy can be attributed to Taiwan having a health care system that prioritizes disease treatment over prevention. Our revisit analysis of the 2017 Global Burden of Disease study revealed that 44.3% of the total disease burden in Taiwan was associated with modifiable risk factors, whereas the leading risk factors in Japan, Singapore, and South Korea had lower fractions of attributable disability-adjusted life year rates of 38.0%, 35.2%, and 40.3%, respectively [52]. The successful reduction in risk exposure and unhealthy behaviors in these countries has led to significant improvements in health outcomes. Our findings clearly indicate that a healthy lifestyle can significantly extend a person’s life span. Our estimates can serve not only as materials for promoting health among the public but also as key references for the government when it seeks to develop, implement, and evaluate intervention programs.

Our study showed that nonsmoking, avoiding excessive alcohol consumption, healthy diet intake, normal weight, and regular physical activity were all associated with a low risk of premature mortality. Nonsmoking and healthy diet intake considerably affected the LE of Taiwanese adults. The harmful effects of smoking are well known. Our findings highlight the importance of maintaining nonsmoking behavior. Healthy diet intake has been associated with low risks of morbidity and mortality associated with several noncommunicable diseases [53]. We did not investigate the effects of the intake of various foods and nutrients; nevertheless, the quantity of fruit and vegetable intake indicates the healthiness level of a diet, at least partially. A significant dose-response relationship was noted between fruit and vegetable intake and an increase in LE. Nevertheless, this relationship has not been found in the burden of health care expenditure. One plausible interpretation is that individuals who adhere to a healthy diet may also exhibit a heightened health consciousness, potentially resulting in excessive use of health care resources, particularly within the environment of Taiwan’s National Health Insurance system. Regarding BMI, individuals who were normal weight or overweight had longer LEs than those who had underweight or obesity. These findings are generally consistent with those of a population-based cohort study, which reported a J-shaped association between BMI and mortality, with the nadir at a BMI of 25 kg/m^2^ [54]. However, the issue of the obesity paradox necessitates thoughtful consideration when using BMI as an indicator of obesity because of its inherent limitations and the potential for misclassification resulting from arbitrary BMI categorization [55-58]. In our study, consistent results were observed when applying both the WHO global and Asian BMI classification systems. Importantly, it should be noted that overweight and obesity exerted significant negative effects, as indicated by the noteworthy excess health care expenditure associated with these conditions. Furthermore, our findings revealed a positive correlation between the level of physical activity and an increase in LE, yet this did not translate into a reduction in health care expenditure. Although physical activity has been associated with improvements in overall LE, engaging in regular exercises (particularly those involving outdoor activities) may increase the risk of accidental injury and thereby lead to additional health care expenditure. Our findings revealed that infrequent alcohol consumption (less than once a week) exerted a favorable effect on LE, as well as health care expenditure. Although the cardioprotective effects of moderate alcohol consumption have been reported in large cohort studies [59], current guidelines do not endorse initiating alcohol consumption solely for the purpose of preventing cardiovascular disease. Notably, a systematic review and meta-analysis reported that low or moderate alcohol consumption was not significantly associated with a reduced risk of all-cause mortality [60]. Furthermore, relying solely on alcohol consumption frequency rather than the actual intake amount (in grams) in this study may result in an imprecise exposure assessment. To address this limitation, we used NHIS 2009 and 2013 data, which began collecting information on alcohol intake amounts, to investigate the relationship between consumption frequency and intake amounts. Our additional analysis revealed a significant correlation, with a Spearman correlation coefficient of 0.986 (Table S6 in Multimedia Appendix 1). However, further research is warranted to refine exposure assessment methods and comprehensively explore the impact of alcohol consumption on LE and health care costs. In addition, there could be notable diversity in the effects on LE and lifetime health expenditures across various combinations of healthy lifestyle factors. Our subgroup analysis reveals that the combination of nonsmoking and adequate fruit and vegetable consumption stands out as the most crucial factors associated with increased LE while maintaining an optimal body weight emerges as pivotal for reducing lifetime health expenditures. However, the constrained sample sizes within the strata might have impeded the detection of discrepancies owing to diminished statistical power. Thus, further investigation with larger samples is imperative to elucidate these potential heterogeneities more definitively.

To the best of our knowledge, this study is the first to quantify the lifetime health care expenditure associated with the individual and combined effects of healthy lifestyle factors. Our results showed that healthy lifestyle factors were associated with reduced annual average health care expenditure. Previous studies exploring the effects of risk behaviors on health care costs have revealed that smoking, excessive alcohol consumption, obesity, and overweight may lead to significant economic burdens [20-22,24-27]. However, less research to date has explored the impact of individual and combined healthy lifestyle factors on lifetime health care expenditure using longitudinal individual data. Adherence to a low-risk lifestyle can not only prolong an individual’s life span but also significantly postpone disability [7,12-17,61]. Li et al [14] reported that individuals aged 50 years who had a low-risk lifestyle had a longer LE free of major chronic diseases, with an approximate gain of 7.6 years for men and 10 years for women in the United States and 6.9 years for men and 9.4 years for women in the United Kingdom population [13]. Furthermore, a recent Chinese study unveiled that adults aged 40 years, who embraced 5 low-risk lifestyle factors, could attain an additional 6.3 and 4.2 years of life without cardiovascular diseases, cancer, and chronic respiratory diseases for men and women, respectively, compared with those with 0 to 1 low-risk lifestyle factors [12]. These findings indicate that postponing disability can benefit society by ensuring that health care costs associated with disability are not incurred until individuals reach advanced ages and enabling individuals to work longer. On the other hand, our investigation has revealed that specific lifestyle factors exhibit disparate effects on both LE and health care expenditures. For example, our findings show that individuals who are overweight (25≤BMI<30 kg/m^2^) experience an extension of 0.69 (95% CI −0.54 to 2.26) years in their LE compared with those with a normal weight (18.5≤BMI<25 kg/m^2^). However, this increased longevity is accompanied by elevated health care costs, resulting in a 21.1% (95% CI 9.98%-31.17%) increase in annual health care expenditure. By carefully considering the trade-offs between the influences of health behaviors on LE and health care expenditure, our outcomes may yield valuable insights for the comprehensive evaluation of cost-effectiveness in primary prevention initiatives. Nonetheless, these findings also emphasize the urgency of future research endeavors to thoroughly investigate the impact of health factors on both nonfatal and fatal disease burdens. For instance, it would be imperative to examine the effects of health factors on composite metrics such as disability-adjusted life years , quality-adjusted life years, or health-adjusted LE at an individual level to gain a comprehensive understanding.

As nations with aging populations face the associated socioeconomic consequences, they must ensure that the health status of older individuals is maintained. Our findings, derived from the Taiwanese population, indicate that significant health and economic gains can be achieved if individuals adopt low-risk behaviors. Taiwan’s Health Promotion Administration has sought to increase the health promotion awareness and the preventive health capabilities of the population. Their efforts have concentrated on the screening of cancer, regulation of tobacco hazards, and prevention of chronic and noncommunicable diseases. These measures may not only reduce disease risks and extend LE but also alleviate economic burdens. For example, consider the tobacco tax revenue in Taiwan for the year 2022, which amounted to approximately 0.97 billion [62]. Approximately 50% (0.49 billion) of this revenue is allocated to subsidize the National Health Insurance expenditure. According to the estimates in this study, combined with a smoking prevalence of 14% in Taiwan [63], we can estimate that approximately an additional 0.36 billion in health care expenditure was contributed by smokers. However, it is important to note that our study does not account for indirect economic impacts, including indirect health care expenditures and productivity losses. In recent decades, Taiwan’s health care expenditures have steadily increased, and the National Health Insurance system currently grapples with a budget deficit. To improve the health care system in Taiwan, adjustments in health promotion investment strategies are necessary. The findings from this study hold significant implications for the direction of health promotion strategies and policy formulation. Previous literature suggests the effectiveness of fiscal policies in improving health, such as the implementation of taxes on tobacco, alcohol, and sugar-sweetened beverages and foods. Nevertheless, it is not limited to taxing substances with health risks. Adjustments may include increasing investments in health education and preventive health services, as well as enhancing food and physical environments to support healthy behaviors.

### Limitations

Our study has several strengths, including the use of a large sample size derived from national representative survey data, a prospective nature, and an almost complete follow-up. By linking these data with National Health Insurance claims and vital registry data and integrating an extrapolated lifetime survival function, we were able to comprehensively evaluate the impacts of health lifestyle factors on LE and health care expenditure in Taiwan. In addition, we integrated IPTW in our survival function extrapolation algorithm to minimize the effects of potential confounders. This innovative approach enhances the robustness and validity of our findings.

This study has several limitations that should be acknowledged. First, all analyzed lifestyle factors were assessed at baseline; therefore, lifestyle changes over time were not accounted for because no repeated measurement data were available. Second, the lifestyle factors data were self-reported, which may have led to misclassification. Third, although we adjusted the statistical models for a wide range of potential confounders, the likelihood of residual and unmeasured confounding effects cannot be ruled out. Fourth, we might have underestimated the proportion of individuals with an unhealthy lifestyle because individuals with poor health may be less likely to participate in surveys or may even die before participating. Fifth, we did not account for other healthy lifestyle factors that may independently affect LE, including factors such as lack of comprehensive dietary intake frequency information (eg, red or processed meat consumption), which prevented us from adequately exploring the impact of various healthy dietary patterns on overall health and economic burden, as well as factors such as sleep quality and duration, and levels of stress. Finally, lifetime health care expenditures were estimated on the basis of reimbursement data obtained from Taiwan’s NHIRD. Therefore, the estimates did not account for out-of-pocket expenses or costs associated with a loss of productivity. Therefore, from a societal perspective, lifetime health care expenditures might have been underestimated in this study.

### Conclusions

Our findings revealed dose-response relationships between healthy lifestyle factors and increased LEs and reduced annual health care expenditures. The findings may have implications for primary prevention and resource allocation. They highlight the need for coordinated multisectorial efforts that target modifiable lifestyle factors to reduce the overall burden of disease. By prioritizing interventions that promote healthy behaviors and mitigate risk factors, health outcomes can be improved and health care costs can be minimized.

## Appendix

Multimedia Appendix 1 Additional methods and results.

## Abbreviations

IPTW: inverse probability of treatment weighting
LE: life expectancy
MET: metabolic equivalent of task
NHIRD: National Health Insurance Research Database
NHIS: National Health Interview Survey
STROBE: Strengthening the Reporting of Observational Studies in Epidemiology
WHO: World Health Organization

## Data Availability

The data used in this analysis are not owned by the authors and therefore cannot be shared publicly. To acquire access to the individual-level data, interested researchers must complete an application form, submit a research proposal, and provide documentation of institutional review board approval to the Health and Welfare Data Science Center, Taiwan [46]. The Center will review these materials and grant access to eligible researchers who meet the criteria for accessing confidential data. It should be noted that authorized researchers will be granted access to the data under the same conditions and procedures as the authors.
