# Supplementary material for: Impact of Healthy Lifestyle Factors on Life Expectancy and Lifetime Health Care Expenditure: Nationwide Cohort Study
Source: JMIR Public Health Surveill. 2024 Jul 17;10:e57045. doi: 10.2196/57045 (PMC11292159; doi:10.2196/57045)
Supplement: Multimedia Appendix 1 [file publichealth_v10i1e57045_app1.docx]

**Impact of Healthy Lifestyle Factors on Life Expectancy and Lifetime Health Care Expenditure: Nationwide Cohort Study**

**Supplementary Methods and Results**

**Contents**

**Supplementary Methods**

**Table S1.** The definitions of comorbid diseases of ICD diagnosis codes

**Empirical validation of survival extrapolation method**

**Table S2.** The validation results for the extrapolation of survival for cohorts with and without risk lifestyle factors

**Supplementary Results**

**Table S3.** Hazard Ratios (HRs) and 95% CIs for associations between healthy lifestyle factors and all-cause mortality risk

**Table S4.** Years of life gained of study cohorts by the number of low-risk lifestyle factors

**Table S5.** Life expectancy, years of life gained, lifetime healthcare expenditure and percentage change in annual healthcare expenditure of study cohorts with different combinations of healthy lifestyle factors within each subgroup

**Table S6.** The mean and quartile levels of alcohol intake (grams per day) by frequency of alcohol consumption

**Figure S1.** Kaplan-Meier plots of each lifestyle factors

**Figure S2.** Scatter diagram of association between life expectancy and lifetime healthcare expenditure

**Figure S3.** Sensitivity analysis of BMI classification defined by WHO Asian BMI risk cut points

**Table S1. The definitions of comorbid diseases of ICD diagnosis codes**

|  | ICD-9-CM | ICD-10-CM |
| --- | --- | --- |
| Cardiovascular diseases (CVD) | 390-398, 410-414, 415-429, 440-459 | I00-I09, I20-I25, I26-I52, I70-I99 |
| Stroke | 430-438 | I60-I69 |
| Diabetes mellitus (DM) | 249, 250, 3620 | E08, E11, E13 |
| Cancer | 140-239 | C00-D49 |
| Asthma or COPD | 491-499 | J41-J45, J47 |
| Chronic kidney disease (CKD) | 585 (403, 404) | N18 (I12, I13) |
| hypertension | 401-405 | I10-I16 |
| hyperlipidemia | 272 | E78 |

**Empirical validation of the adjusted rolling extrapolation algorithm**

The validation of the proposed method was performed on the weighted survival times of the cohorts. For each cohort, we created a data set of survival times with the assumption that the participants were followed 160 months. The censoring rates of these data sets ranged from 80% and 89%. We applied the proposed method to these data sets to extrapolate the survival for the next 60 months. Because these cohorts were actually followed more than 220 months, the true estimates of the survival rates during the extended period of 5 years for the cohorts can be considered as the benchmark for comparison. The mean absolute relative error, denoted by $\bar{E},$ was defined by averaging the absolute difference between extrapolated survival rates and the true rates and divided by the true ones during the 60 months of extrapolation. For each cohort, we also used bootstrap method to generate 100 data sets and calculate absolute relative error of the extrapolated result on each bootstrap sample.

Table S2 gave the censoring rates, mean absolute relative errors, minimum and maximum of the mean absolute relative errors from 100 bootstrap samples. We successfully demonstrated an exceptionally precise estimation of the projected survival rate at the ensuing $60$-month period for the cohorts with and without risk lifestyle factors, yielding an absolute relative error ranged from 0.27% to 2.09%. These findings highlight the ability of rolling extrapolation algorithms to provide accurate survival predictions, with relative bias well controlled within acceptable thresholds.

**Table S2. The validation results for the extrapolation of survival for cohorts** **with and without risk lifestyle factors**

| Lifestyle Factor |  | $\mathbf{censoring}$  $\mathbf{rate}$ | $\bar{\boldsymbol{E}}\boldsymbol{(\%)}$ | $\mathbf{SD}_{\bar{\boldsymbol{E}}}$ | $\mathbf{Min}_{\bar{\boldsymbol{E}}}$ | $\mathbf{Max}_{\bar{\boldsymbol{E}}}$ |
| --- | --- | --- | --- | --- | --- | --- |
| Cigarette smoking | Never smoker | 0.865 | 0.27 | 0.38 | 0.16 | 1.97 |
|  | Current or former smokers | 0.831 | 1.31 | 0.88 | 0.21 | 3.92 |
| Alcohol consumption | Infrequent or non-consumers | 0.863 | 0.55 | 0.28 | 0.15 | 1.51 |
|  | Excess consumers | 0.868 | 1.86 | 0.77 | 0.20 | 3.65 |
| Leisure-time physical activity | Sufficient active | 0.876 | 0.88 | 0.50 | 0.09 | 2.45 |
|  | Insufficient active | 0.847 | 0.44 | 0.38 | 0.13 | 1.76 |
| Fruit and vegetable intake | Sufficient intake | 0.866 | 1.11 | 0.40 | 0.12 | 1.92 |
|  | Low or insufficient intake | 0.815 | 2.09 | 1.05 | 0.21 | 5.05 |
| BMI group | Optimal body weight | 0.853 | 0.71 | 0.33 | 0.08 | 1.44 |
|  | Non-optimal body weight | 0.860 | 0.42 | 0.41 | 0.21 | 2.00 |
|  |  |  |  |  |  |  |
| No. of low-risk factors | 0 or 1 | 0.808 | 0.46 | 1.06 | 0.36 | 4.17 |
|  | 2 | 0.799 | 0.37 | 0.82 | 0.24 | 5.00 |
|  | 3 | 0.851 | 0.29 | 0.52 | 0.15 | 3.00 |
|  | 4 | 0.865 | 0.62 | 0.54 | 0.23 | 3.23 |
|  | 5 | 0.889 | 1.74 | 0.85 | 0.22 | 3.83 |

$\mathrm{SD}_{\bar{E}}$, $\mathrm{Min}_{\bar{E}},$ and $\mathrm{Max}_{\bar{E}}$ are standard deviation, minimum value and maximum value of the mean absolute relative errors ($\bar{E}$) from 100 bootstrapped estimates

**Table S3. Hazard Ratios (HRs) and 95% CIs for associations between healthy lifestyle factors and all-cause mortality risk**

|  |  |  | **Model 1^a^** | | **Model 2^b^** | |
| --- | --- | --- | --- | --- | --- | --- |
| **Lifestyle factors** | **Deaths (%)** | **Person-years** | **HR (95%CI)** | | **HR (95%CI)** | |
| Cigarette smoking |  |  |  |  |  |  |
| Never smokers | 16.41 | 219,690 | 1.00 |  | 1.00 |  |
| Current or former smokers | 25.12 | 97,426 | 1.40 | (1.29, 1.51) | 1.36 | (1.26, 1.48) |
| Alcohol consumption |  |  |  |  |  |  |
| Infrequent or nonconsumers | 18.60 | 264,386 | 1.00 |  | 1.00 |  |
| Excess consumers | 22.06 | 52,729 | 1.23 | (1.13, 1.34) | 1.22 | (1.12, 1.33) |
| Leisure-time physical activity |  |  |  |  |  |  |
| Sufficient active | 21.16 | 106,716 | 1.00 |  | 1.00 |  |
| Insufficient active | 18.16 | 210,400 | 1.26 | (1.18, 1.34) | 1.18 | (1.11, 1.27) |
| Fruit and vegetable intake |  |  |  |  |  |  |
| Sufficient intake | 17.79 | 285,011 | 1.00 |  | 1.00 |  |
| Low or insufficient intake | 30.64 | 32,105 | 1.48 | (1.36, 1.62) | 1.37 | (1.25, 1.49) |
| BMI group |  |  |  |  |  |  |
| Normal weight | 18.21 | 198,203 | 1.00 |  | 1.00 |  |
| Underweight | 27.38 | 11,911 | 1.60 | (1.39, 1.83) | 1.71 | (1.49, 1.97) |
| Overweight | 19.95 | 107,002 | 1.05 | (0.98, 1.12) | 0.94 | (0.87, 1.00) |
| No. of low-risk lifestyle factors |  |  |  |  |  |  |
| 0 | 28.49 | 2,854 | 1.00 |  | 1.00 |  |
| 1 | 25.15 | 15,847 | 0.77 | (0.57, 1.03) | 0.85 | (0.63, 1.14) |
| 2 | 24.00 | 45,489 | 0.56 | (0.42, 0.74) | 0.62 | (0.47, 0.82) |
| 3 | 20.44 | 90,559 | 0.44 | (0.33, 0.58) | 0.51 | (0.39, 0.68) |
| 4 | 16.41 | 116,330 | 0.35 | (0.26, 0.46) | 0.43 | (0.32, 0.57) |
| 5 | 15.82 | 46,037 | 0.27 | (0.20, 0.36) | 0.37 | (0.27, 0.49) |

^a^model 1 adjusted for age, sex, enrolment year, and five lifestyle factors;

^b^model 2 adjusted for age, sex, enrolment year, ethnicity, education level, marital status, religion, monthly household income, and medical history of hypertension, hyperlipidemia, cardiovascular disease, diabetes mellitus, cancer, chronic lung diseases, and chronic kidney diseases, and five lifestyle factors in the table.

**Table S4. Years of life gained of study cohorts by the number of low-risk lifestyle factors**

| **No. of low-risk lifestyle factors** | **N** | **LE** | **(95%CI)** | **Years of life gained** | **(95%CI)** |
| --- | --- | --- | --- | --- | --- |
| 0 | 186 | 24.19 | (20.03, 31.73) | Reference |  |
| 1 | 1,034 | 28.56 | (24.05, 33.37) | 4.37 | (-3.06, 11.13) |
| 2 | 2,929 | 28.92 | (24.92, 33.07) | 4.73 | (-3.12, 10.85) |
| 3 | 5,748 | 34.41 | (33.81, 36.02) | 10.22 | (3.15, 15.05) |
| 4 | 7,183 | 36.04 | (34.10, 36.79) | 11.85 | (4.19, 16.18) |
| 5 | 2,813 | 36.58 | (34.22, 38.57) | 12.39 | (5.71, 17.64) |

**Table S5. Life expectancy, years of life gained,** **lifetime healthcare expenditure** **and percentage change in annual healthcare expenditure of study cohorts with different combinations of healthy lifestyle factors within each subgroup**

| **Lifestyle factors^*^** | **N** | **LE** | **(95%CI)** | **Years of life gained** | **(95%CI)** | **Lifetime healthcare expenditure, dollars^#^** | **(95%CI)** | **% change in annual healthcare expenditure**^¶^ | **(95%CI)** |
| --- | --- | --- | --- | --- | --- | --- | --- | --- | --- |
| **0 or 1 low-risk lifestyle factors** | 1,220 | 29.19 | (25.45, 33.62) | Reference |  | 58,715 | (43,229, 78,470) | Reference |  |
| **2 low-risk lifestyle factors** | 2,929 | 30.69 | (26.25, 32.76) | 1.50 | (-5.28, 6.36) | 53,414 | (45,675, 58,903) | -16.01% | (-46.06, 12.46) |
| NA+FV | 914 | 31.78 | (25.45, 33.22) | 2.59 | (-5.61, 6.73) | 58,693 | (47,406, 66,966) | -9.75% | (-37.72, 19.13) |
| FV+OW | 780 | 32.32 | (27.78, 35.28) | 3.13 | (-3.88, 8.89) | 45,629 | (36,648, 53,464) | -35.44% | (-67.80, -8.48) |
| NA+OW | 289 | 27.73 | (21.88, 32.16) | -1.46 | (-10.44, 5.30) | 46,530 | (33,543, 60,719) | -19.67% | (-56.43, 18.42) |
| NS+FV | 285 | 34.67 | (27.37, 39.57) | 5.49 | (-4.21, 12.17) | 65,205 | (41,069, 95,392) | -7.74% | (-58.24, 55.69) |
| NS+NA | 279 | 28.13 | (25.18, 31.08) | -1.06 | (-6.95, 4.59) | 53,385 | (39,300, 66,948) | -6.73% | (-40.97, 33.81) |
| PA+FV | 215 | 29.93 | (25.34, 32.52) | 0.75 | (-6.06, 6.20) | 63,477 | (47,501, 79,812) | 6.44% | (-27.36, 44.01) |
| **3 low-risk lifestyle factors** | 5,748 | 34.42 | (33.82, 36.04) | 5.24 | (0.98, 9.70) | 58,246 | (40,663, 63,068) | -18.90% | (-46.53, -1.70) |
| NS+NA+FV | 2,576 | 35.68 | (33.40, 36.88) | 6.49 | (0.71, 10.37) | 66,162 | (60,024, 72,997) | -9.27% | (-36.24, 22.88) |
| NA+FV+OW | 1,392 | 34.32 | (32.76, 36.19) | 5.13 | (0.08, 9.86) | 53,694 | (38,532, 76,571) | -26.40% | (-67.18, 22.09) |
| NA+PA+FV | 451 | 31.13 | (27.89, 32.97) | 1.94 | (-4.02, 6.40) | 55,690 | (46,261, 64,945) | -13.17% | (-48.39, 25.37) |
| NS+NA+OW | 421 | 29.11 | (25.92, 33.29) | -0.07 | (-6.05, 7.08) | 32,545 | (27,673, 40,553) | -52.81% | (-81.66, -31.94) |
| NS+FV+OW | 334 | 39.83 | (38.12, 41.56) | 10.64 | (5.77, 14.83) | 70,475 | (49,253, 89,970) | -14.29% | (-59.34, 28.31) |
| PA+FV+OW | 258 | 27.26 | (24.48, 29.26) | -1.92 | (-7.74, 2.40) | 36,826 | (29,572, 45,726) | -39.04% | (-72.46, -9.87) |
| NS+PA+FV | 141 | 35.50 | (33.37, 39.27) | 6.31 | (0.24, 11.92) | 56,441 | (41,783, 70,877) | -24.93% | (-58.72, 7.03) |
| **4 low-risk lifestyle factors** | 7,183 | 35.77 | (34.27, 36.83) | 6.58 | (1.13, 10.76) | 60,667 | (40,685, 67,255) | -18.61% | (-47.41, -1.84) |
| NS+NA+FV+OW | 4,675 | 39.09 | (37.57, 40.39) | 9.91 | (5.51, 14.07) | 52,717 | (48,511, 56,810) | -39.16% | (-69.24, -18.79) |
| NS+NA+PA+FV | 1,527 | 33.54 | (31.92, 37.94) | 4.36 | (0.03, 10.56) | 67,677 | (62,023, 76,016) | 0.35% | (-33.44, 32.26) |
| NA+PA+FV+OW | 668 | 29.86 | (27.83, 31.74) | 0.67 | (-4.78, 5.45) | 54,128 | (44,610, 65,106) | -11.75% | (-51.44, 19.55) |
| NS+PA+FV+OW | 205 | 33.96 | (29.09, 36.23) | 4.77 | (-2.12, 9.57) | 51,151 | (34,205, 67,048) | -29.83% | (-64.96, 10.18) |
| NS+NA+PA+OW | 108 | 28.34 | (22.90, 32.27) | -0.85 | (-8.6, 5.15) | 44,100 | (30,887, 57,272) | -26.88% | (-67.62, 8.20) |
| **5 low-risk lifestyle factors** | 2,813 | 36.32 | (32.76, 38.42) | 7.13 | (1.33, 11.11) | 55,785 | (47,896, 64,346) | -28.12% | (-57.61, -4.43) |

LE: life expectancy; NS: Nonsmoking, NA: Avoiding excessive alcohol consumption, PA: Sufficient physical activity, FV: Sufficient fruit and vegetable intake, OW: Maintaining an optimal body weight.

* Only sub-cohorts with more than 100 participants were included in the analysis.

# $1 dollar (US) = $30.65 dollars (New Taiwan).

¶ The percentage change in annual healthcare expenditure was calculated as the difference in per capita annual healthcare expenditure between population with and without healthy lifestyle factors, divided by the annual average healthcare expenditure per capita for the overall population.

**Table S6. The mean and quartile levels of alcohol intake (grams per day) by frequency of alcohol consumption**

|  |  | Amounts of alcohol intake (grams per day) | | | | |
| --- | --- | --- | --- | --- | --- | --- |
| Alcohol consumption (frequency) | **N** | **Mean** | **SD** | **Q1** | **Q2** | **Q3** |
| Never drinkers | 14,483 | 0.00 | 0.00 | 0.00 | 0.00 | 0.00 |
| Infrequent drinkers | 4,287 | 1.77 | 3.69 | 0.28 | 0.59 | 1.76 |
| Regular drinkers | 2,996 | 28.20 | 69.58 | 4.06 | 11.17 | 28.44 |
| Heavy drinkers | 584 | 70.69 | 112.71 | 13.10 | 33.18 | 85.32 |

Spearman correlation coefficient $\rho=0.986 (p value<0.0001)$

Data source: NHIS 2009 & 2013.

**
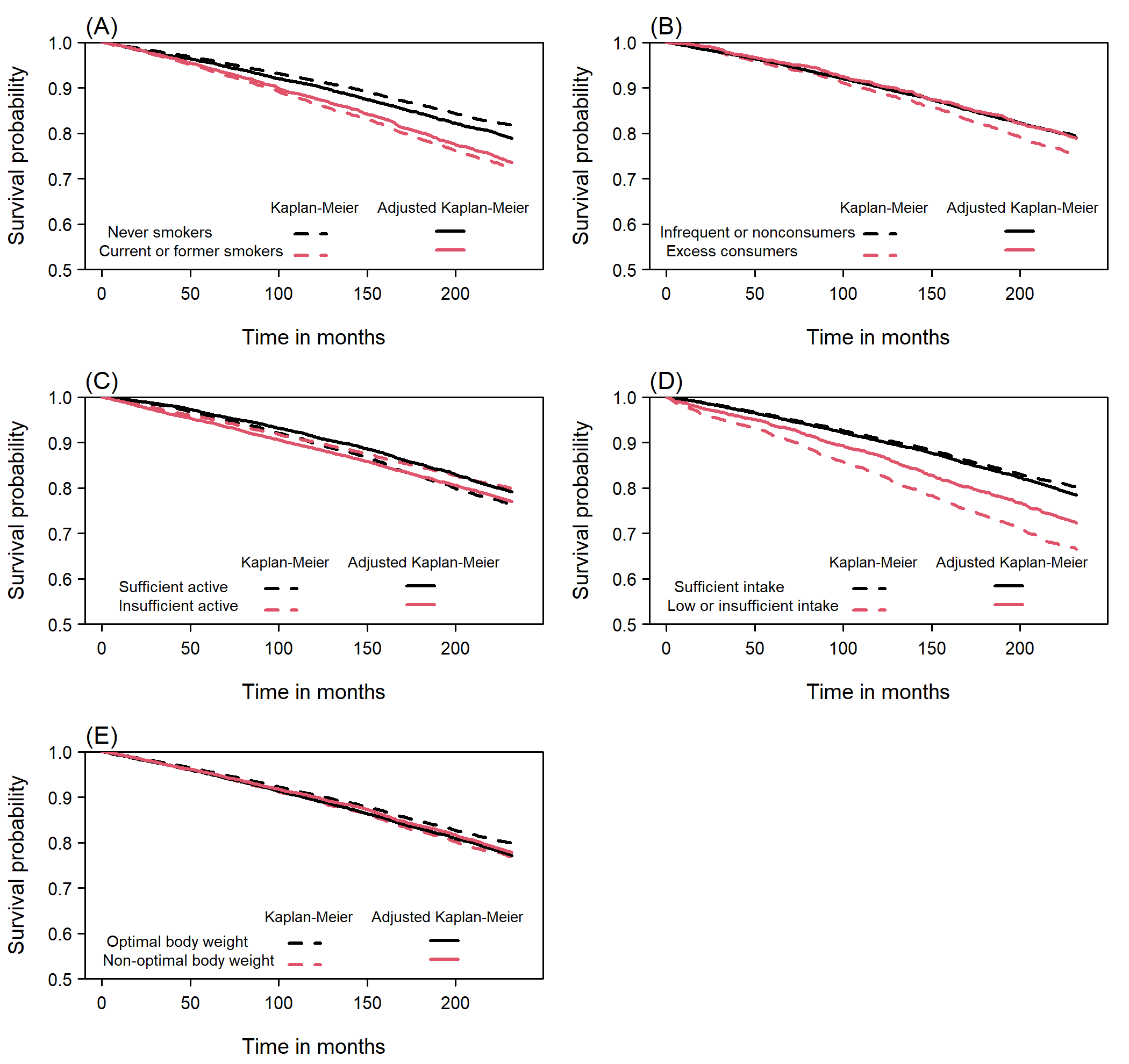
**

**Figure S1. Survival curves of each lifestyle factor during the follow-up estimated by Kaplan-Meier estimator and adjusted Kaplan-Meier estimator. (A) Cigarette smoking, (B) Alcohol consumption, (C) Leisure-time physical activity, (D) Fruit and vegetable intake, and (E) BMI group**


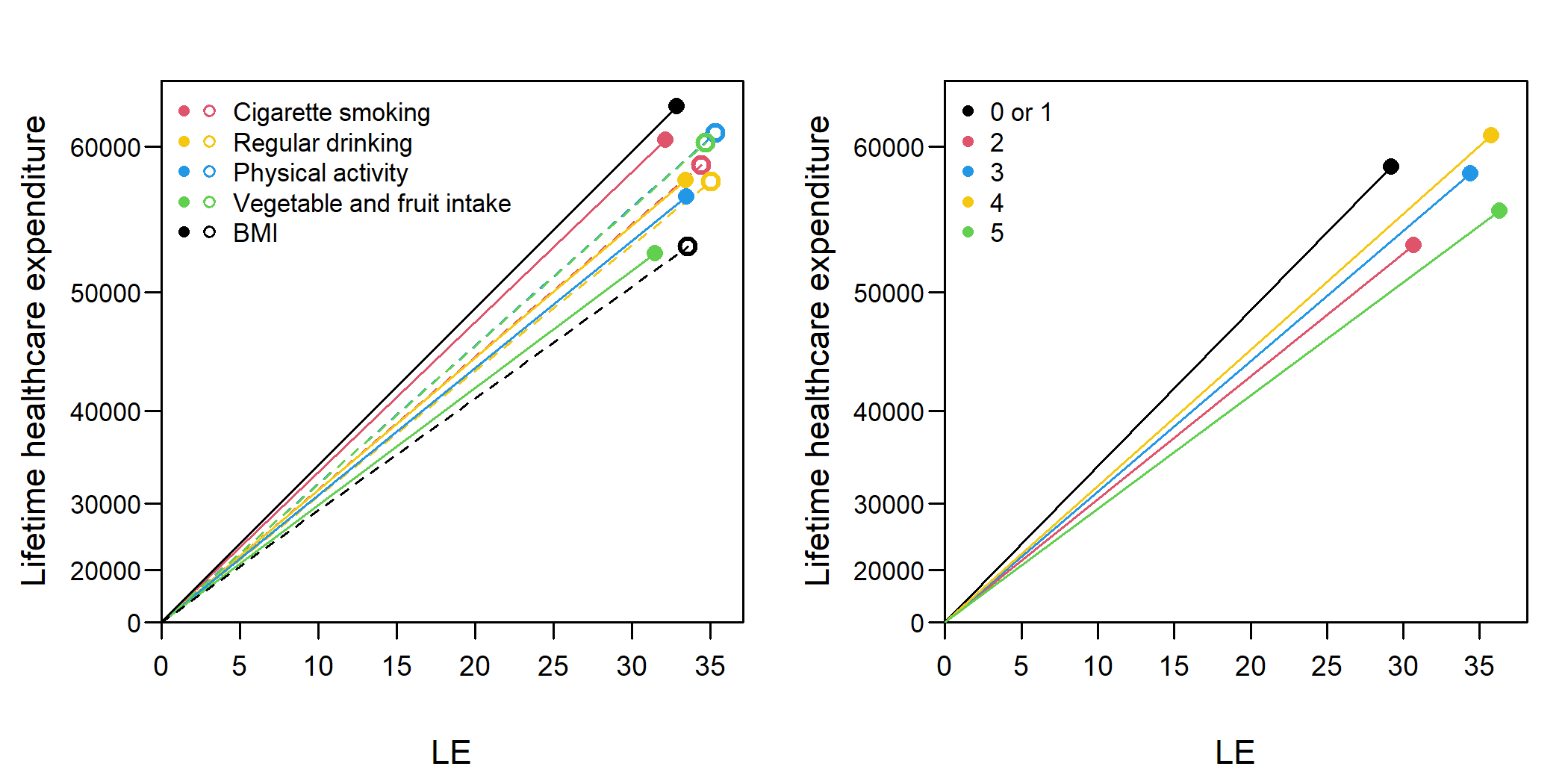


(B)

(A)

**Figure S2. Scatter diagram of association between life expectancy and lifetime healthcare expenditure.** (A) classified by the presence or absence of healthy lifestyle factors (empty circles represent population with healthy lifestyle factors; filled circles represent population without healthy lifestyle factors), and (B) categorized by the number of healthy lifestyle factors.


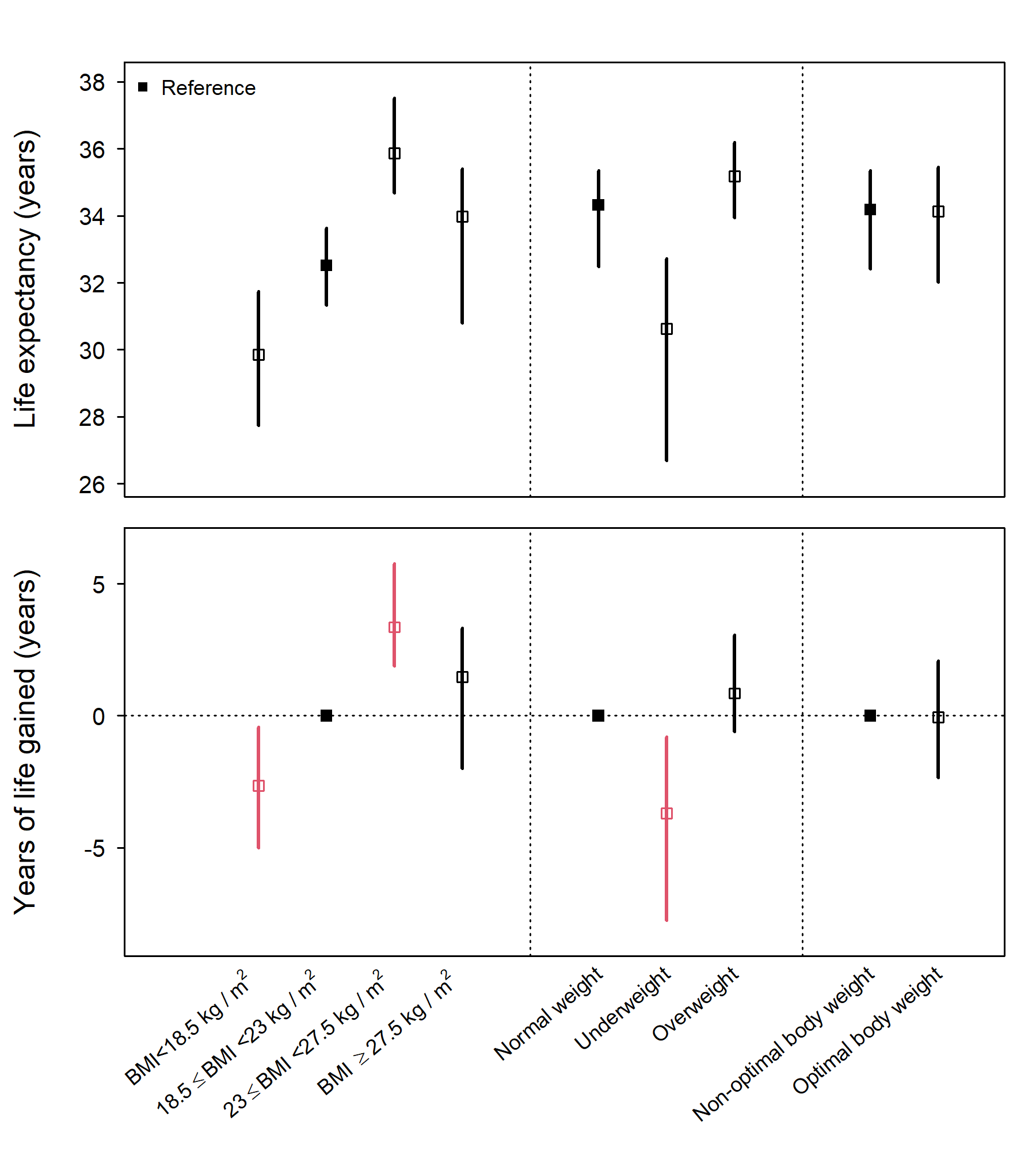


**Figure S3. Sensitivity analysis of BMI classification defined by WHO Asian BMI risk cut points.** BMI: body mass index. Optimal body weight (normal weight): 18.5 ≤ BMI < 23 kg/m^2^; non-optimal body weight includes underweight (BMI <18.5 kg/m^2^) and overweight (BMI ≥ 23 kg/m^2^).

Red color indicates the estimated value was significantly higher or lower than reference group.
